# Comparative Study of Bioactive Compounds and Biological Activities of Five Rose Hip Species Grown in Sicily

**DOI:** 10.3390/plants13010053

**Published:** 2023-12-23

**Authors:** Barbara Guantario, Nicoletta Nardo, Giancarlo Fascella, Giulia Ranaldi, Paola Zinno, Alberto Finamore, Gianni Pastore, Michele Massimo Mammano, Irene Baiamonte, Marianna Roselli

**Affiliations:** 1 CREA-Research Centre for Food and Nutrition, Via Ardeatina, 546, 00178 Rome, Italy; barbara.guantario@crea.gov.it (B.G.); nicoletta.nardo@crea.gov.it (N.N.); giulia.ranaldi@crea.gov.it (G.R.); alberto.finamore@crea.gov.it (A.F.); giovanni.pastore@crea.gov.it (G.P.); 2 CREA-Research Centre for Plant Protection and Certification, Viale delle Scienze, Building 4, 90128 Palermo, Italy; giancarlo.fascella@crea.gov.it (G.F.); massimo.mammano@crea.gov.it (M.M.M.); 3 Institute for the Animal Production System in the Mediterranean Environment (ISPAAM), National Research Council, Piazzale Enrico Fermi 1, 80055 Portici, Italy; paola.zinno@cnr.it

**Keywords:** *Rosa* spp., rose hips, phenolic profile, vitamin C, carotenoids, intestinal permeability, Caco-2 cells

## Abstract

Nowadays, research on plant extracts has attracted increasing interest. The aim of this study was to compare phenolic profile, vitamin C, and carotenoid content, as well as the biological activities of five different rose species, including *Rosa canina*, *R. corymbifera*, *R. micrantha*, *R. rubiginosa,* and *R. rugosa*. These species had different morphological characteristics, with *R. rugosa* showing higher size of flower petals and higher weight of hips. The highest vitamin C content was found in hip extracts of *R. rubiginosa* and *R. rugosa*, which also showed the highest carotenoid amount. *R. corymbifera* showed the highest phenolic content. No significant antimicrobial activity of extracts containing phenolic compounds against different indicator strains could be detected. Cell monolayer integrity was not affected by treatments with the above-mentioned extracts of *R. canina*, *R. micrantha,* and *R. rugosa* at different concentrations for up to 24 h, while those of *R. rubiginosa* and *R. corymbifera* affected intestinal permeability at the highest concentration tested. The partial least squares regression analysis generated a predictive model correlating phenolic compounds with cell monolayer integrity, suggesting a relevant role for catechin, quercitrin, and p-coumaric acid. In conclusion, this study highlights how rose hips belonging to different species can have a diverse phenolic profile, differently influencing intestinal monolayer integrity.

## 1. Introduction

The relationship between diet and health has been demonstrated by numerous studies, and for this reason, the interest in new functional foods and new nutraceuticals has been increasing. These foods, besides being a source of nutrients, can bring an overall improvement in physical well-being and can contribute to counteracting many diseases, such as cardiovascular disease or diabetes, or to slowing down the ageing process [1]. In recent years, consumers have increased their interest in nutraceuticals and plant-based food supplements, and a great deal of research has turned to raw materials of plant origin that are very rich in biologically active molecules. Plants, in fact, produce a large variety of secondary metabolites, phenolic compounds, and carotenoids, which are of particular interest in the pharmaceutical and food fields. The concentration of bioactive compounds is influenced by agricultural practices, environmental and genetic factors, as well as stress [2].

Rose hips are the accessory fruits of various *Rosa* species [3,4]. Traditionally, in many countries, these pseudo-fruits have been harvested for food or medicinal purposes [5]. Several studies have reported that rose hips are rich in biologically active molecules such as anthocyanins, ascorbic acid, and phenolic compounds [5,6]. These bioactive compounds contribute to the nutritional quality of the plant [6] and can exert a positive effect on health thanks to their antioxidant and antimutagenic activities, through which they can contribute to the prevention of cancer cell proliferation and cardiovascular diseases [7,8,9]. Moreover, rose hips are also used to cure illnesses such as influenza and other infections, inflammatory diseases, chronic pain, and ulcers [10]. Despite species variation, rose hips contain about 20- to 30-fold more vitamin C compared to oranges. Therefore, rose hip fruits are widely used in food and pharmaceutical industries and are added to probiotic beverages, fruit yoghurts, and soups [5]. The *Rosa* genus comprises nearly 200 species with complex taxonomy [11]. Roses are widespread in temperate to subtropical habitats of Europe, Asia, the Middle East, and North America. Rose hips are found in different sizes and colours, from yellow–orange to dark red and sometimes even black, depending on the pattern of pigments such as carotenoids, flavonoids, or anthocyanins [12,13].

Sicily (South Italy) is located at the centre of the Mediterranean basin and is commonly considered by ecologists and botanists to be a biodiversity hotspot [14]. Several *Rosa* species are native to Sicilian spontaneous flora, while others coming from different regions and countries are acclimatised and well-adapted to the south Mediterranean climate [15]. *Rosa canina* L., *R. corymbifera* Borkh., *R. micrantha* Borrer ex Sm, and *R. rubiginosa* L. are up to 3 m tall bushes with arching prickly branches, pale rose flowers, and oblong or sub-spherical red hips, commonly used in phytomedicine and as food component, and all are typical of the vascular flora of Sicily [16]. Differently from these latter, *R*. *rugosa* Thunb. (a.k.a. wrinkled rose or Japanese rose) is native to Eastern Asia (Far East countries) and is characterised by erect, tomentose, and densely prickly branches, fragrant purplish-pink or white flowers, and globose, smooth hips; though of Asian origin, it was introduced to North America and Europe in the nineteenth century as an ornamental plant and can now be found in numerous public and private gardens, as well as in marginal and coastal areas [17]. Up to now, only one study was conducted by Fascella et al., 2019a [8] on the biochemical characterisation of four Sicilian rose hip species, while no studies are available in the literature about the phytochemical properties of hips from *R*. *rubiginosa* and *R. rugosa* plants grown in Sicily. Therefore, the aim of the present work was to determine and compare the phenolic profile, the vitamin C, and the carotenoid content, as well as the biological activities of five rose hip species, including *R*. *canina*, *R. corymbifera*, *R. micrantha*, *R. rubiginosa,* and *R. rugosa* cultivated in Sicily under the same growing conditions. The biological properties were assayed as antimicrobial activity against various pathogen indicator strains, while the effect on intestinal barrier integrity was evaluated in a largely employed in vitro model of human intestinal epithelial cells, the Caco-2 cell line. Indeed, these cells, derived from a colorectal adenocarcinoma, are one of the most characterised intestinal cell lines that can differentiate in vitro and achieve the morpho-functional characteristics of mature enterocytes, with brush border microvilli and epithelial cellular junctions, thus reproducing the organisation and function of the intestinal mucosa barrier, where the apical surface corresponds to the intestinal lumen and the basolateral to the basal lamina [18].

## 2. Results and Discussion

### 2.1. Morphological Characteristics of Rose Leaves, Flowers, and Hips

**Figure 1:**
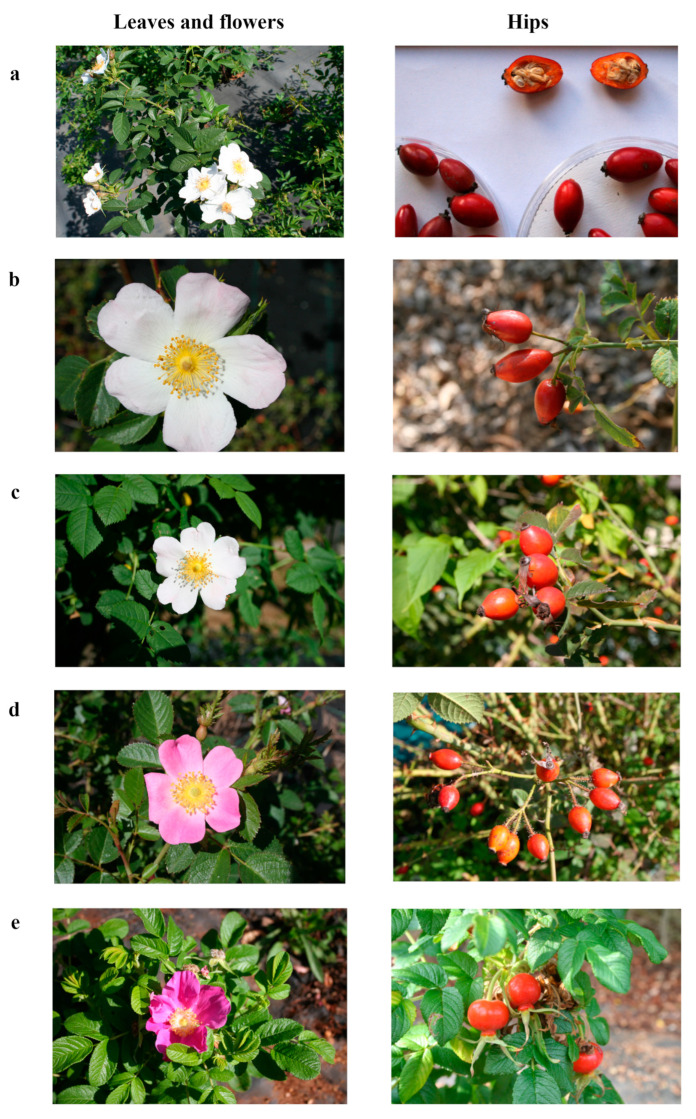
Representative images of leaves, flowers (left panels), and hips (right panels) of *Rosa canina* (**a**), *R. corymbifera* (**b**), *R. micrantha* (**c**), *R. rubiginosa* (**d**), and *R. rugosa* (**e**).

The main morphological characteristics of the five studied rose species are reported in Table 1 and shown in Figure 1. The highest number of leaflets/leaves was recorded on *R. rugosa* plants, whereas lower values were recorded on *R. micrantha* and *R. rubiginosa* plants. The leaflet length was higher in *R. canina* and lower in *R. rubiginosa*. *R. canina* was also characterised by higher leaflet width, while *R. rubiginosa* showed a lower value. The internode length ranged from 3.7 cm to 1.6 cm for *R. corymbifera* and *R. rugosa*, respectively (Table 1). The petal colour of *R. canina*, *R. corymbifera,* and *R. micrantha* flowers turned from pale pink to white during blooming time (Figure 1a–c), whereas petals from *R. rubiginosa* and *R. rugosa* were intense pink-coloured and purple, respectively (Figure 1d,e). The petal length was higher in *R. rugosa* flowers and lower in *R. micrantha* ones. Similarly, the petal width was higher in *R. rugosa* flowers and lower in *R. corymbifera* ones. The hypanthium width was higher in *R. rugosa* flowers and lower in the other species. These outcomes agree with those reported by other studies [8,16], confirming the effect of the genotype on the biometrical features of rose plants under the same climatic conditions and growing techniques. *R. canina* and *R. corymbifera* showed oblong hips (Figure 1a,b); *R. micrantha* and *R. rubiginosa* produced sub-globose hips (Figure 1c,d), while *R. rugosa* showed globose pseudo-fruits (Figure 1e). Limited differences were recorded among the five species regarding hip length as well as hip width. The hip weight was higher in *R. rugosa* and lower in *R. canina*. *R. rugosa* was characterised by a higher number of seeds/hips, whereas lower seed production was recorded in *R. micrantha* hips (Table 1).

### 2.2. Vitamin C Content

**Figure 2:**
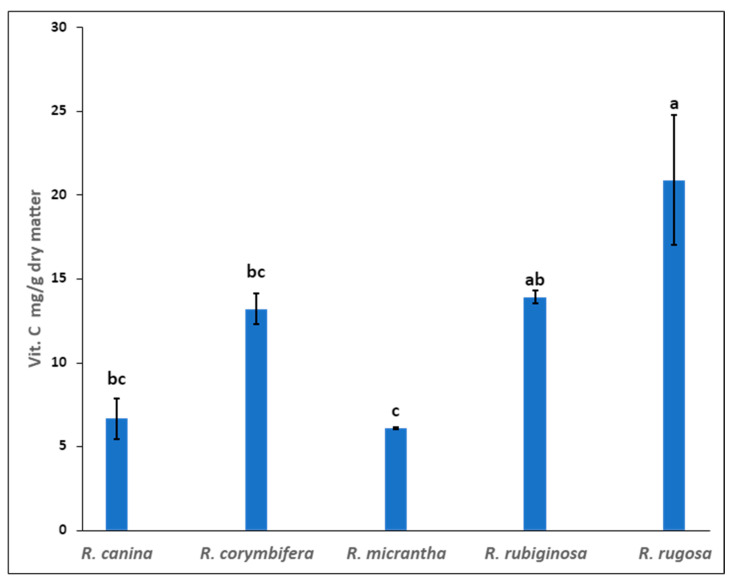
Vitamin C content (ascorbic acid AA and dehydroascorbic acid DHAA mg/g dry matter) in the five rose hip species grown in Sicily. Results are expressed as mean ± standard deviation of two independent extractions. Values with different letters significantly differ (*p* < 0.05).

Rose hips are a valuable natural source of vitamin C. Moisture content of fresh rose hips was determined before chemical analysis (Supplementary Table S1). The vitamin C content of the five rose hip species is presented in Figure 2. The highest vitamin C content was found in hip extracts of *R. rugosa* and *R. rubiginosa* (20.9 and 13.9 mg/g dry matter, respectively), while the lower content of ascorbic acid was found in hip extracts of *R. micrantha* (6.1 mg/g dry matter). Rose hips are generally harvested once a year (in autumn) when they reach full ripening and, consequently, a high content of carotenoids, flavonoids, and vitamin C. It appears from the literature that the ripening degree and the harvest period significantly determine the composition profile of bioactive compounds [19]. Some authors suggested that the level of vitamin C in rose hips could depend on the soil and climate conditions [20,21]. The influence of the genotype on the ascorbic acid content of the rose hips shown in our study seems to be confirmed by other authors [5,8]. The vitamin C content in rose hips has been reported as being quite variable, ranging from 180 to 965 mg/100 g fresh weight [22,23], depending on the species and cultivar. In recent years, several species of rose have been studied because they are a good source of bioactive compounds, and it has also been observed that the high content of carotenoids and flavonoids in rose hips prevents the oxidation of vitamin C, thus increasing its stability and bioavailability [21].

### 2.3. Phenolic Compound Content

Recent developments in the fields of health and food have led to a renewed interest in natural compounds with antioxidant potential. A diet rich in antioxidant components has potential beneficial effects on human health by reducing the risk of various diseases, for example, cardiovascular diseases, cancers, and age-related macular degeneration [24]. The selection of species/varieties with high contents of bioactive compounds and harvesting at the optimum time can promote the uptake increase in bioactive compounds from fruits and vegetables [25]. Polyphenols are secondary metabolites of plants, widely distributed in fruits, vegetables, and plant-derived foods [26]. Some previous studies have confirmed the presence of phenolic compounds in rose hips [27]. In the five species of rose analysed in the present study, seven different phenolic compounds were identified (Table 2). This analysis showed that while the qualitative composition of examined phenolics in the extracts was similar, quantitative differences among the species were evident. The highest phenolic content was found in *R. corymbifera* and *R. canina*, while the lowest phenolic content was found in *R. micrantha*. The amounts of individual phenolic compounds ranged from 0.001 mg/g of dry matter (quercetin) to 0.419 mg/g of dry matter (catechin), as shown in Table 2. Differences in the concentration of phenolic compounds in rose hips among various species were also found by Demir et al. [5]. Several studies also reported a diversified phenolic profile among the various rose species. In the five species of rose analysed in our study, among the phenolic acids, small traces of p-coumaric acid could be found. Indeed, flavonoids, including catechin, rutin, quercetin 3-O-glucoside (isoquercetin), kaempferol 3-O-rutinoside (nicotiflorin), quercitrin, and quercetin, were determined in different quantities among the species. The values for catechin and rutin, the main flavonoids, ranged from 0.006 to 0.419 mg/g of dry matter and from 0.025 to 0.125 mg/g of dry matter, respectively. The accumulation of phenolic compounds in plants can be affected by different factors, such as harvesting season, yearly variance, altitude, plant genotype [4], environmental factors (e.g., light, temperature, soil nutrients), maturity stages of the hips, as well as by various stresses. All these factors may affect the metabolism and conversions of phenolics and could explain the dissimilarities reported among scientific studies [5,25,28].

### 2.4. Carotenoids Content

Carotenoids are important antioxidants and valuable bioactive compounds contributing to the health benefits of different foods. Rose hips are generally known to contain high levels of carotenoids; differences in content may exist due to genetic variation, degree of ripening, growing and storage conditions, and analytical method of extraction [19]. Many of the carotenoids commonly found in rose hips, such as zeaxanthin, lutein, lycopene, and β-carotene, have been shown to have health-beneficial effects [29]. Four main carotenoids (lutein, zeaxanthin, β-carotene, and lycopene) were identified in the rose hips analysed in the present study (Table 3). The highest carotenoid content was found in *R. rugosa* (sum = 135.13 µg/g dry matter), while the lowest carotenoid content was found in *R. canina* (sum = 41.63 µg/g dry matter). The amounts of individual carotenoids ranged from 1.42 µg/g of dry matter (zeaxanthin) to 80.96 µg/g of dry matter (lycopene), as shown in Table 3. As reported by previous studies, our data also show variability in the content of carotenoids in different species [8,19,29].

### 2.5. Evaluation of Biological Activity of Rosa Hip Extracts Containing Phenolic Compounds

To evaluate potential antimicrobial activity, extracts containing phenolic compounds (resuspended in ethanol:water 80/20 *v*/*v*, as well as in PBS) from the five *Rosa* species were tested against different pathogen and alternative bacterial strains, both Gram-positive (*Listeria* strains) and negative (*Salmonella*, *Escherichia,* and *Pseudomonas* strains). The agar spot test performed in the present study did not show any significant activity, as no inhibition halos could be detected at both tested concentrations, regardless of the analysed bacteria (Supplementary Table S2). There are some studies in the literature describing the antimicrobial and antioxidant effects of flower extracts from certain rose varieties [30,31], which can be attributed to the presence of high concentrations of quercetin and kaempferol and their derivatives. According to Cendrowski et al. [32], the aqueous extract of rose hips was particularly effective in inhibiting the growth of bacteria, particularly Gram-positive bacteria, with greater sensitivity shown by the *Bacillus cereus* strain, and Gram-negative bacteria, with greater sensitivity shown *Escherichia coli* and *Klebsiella pneumoniae*. However, it should be noted that the reduction in the number of bacterial cells in the above-mentioned study was influenced by the concentration of the aqueous extract used, but no complete elimination of bacterial growth was observed at any of the concentrations tested. Thus, there is currently insufficient data in the scientific literature to support the hypothesis of antimicrobial activity of rose hip extracts against pathogens.

The potential application of *Rosa* hip extracts in food and nutraceutical fields should be preceded by an assessment of their potential adverse and beneficial effects on intestinal health. Thus, in the present study, the impact of the five extracts on human intestinal Caco-2 cells has been investigated since the intestinal mucosa represents an important site of contact between ingested food and the organism. To evaluate if exposure to *Rosa* extracts containing phenolic compounds could perturb intestinal epithelial permeability, Transepithelial Electrical Resistance (TEER) and phenol red passage (apparent permeability, Papp) were measured in differentiated Caco-2 cells following apical treatment with several concentrations (ranging from 1 to 25 mg/mL) of the five extracts for up to 24 h. An amount of 2% ethanol, corresponding to the concentration contained in the higher dilution of tested extracts, did not affect cell permeability for up to 24 h, as already shown [33].

**Figure 3:**
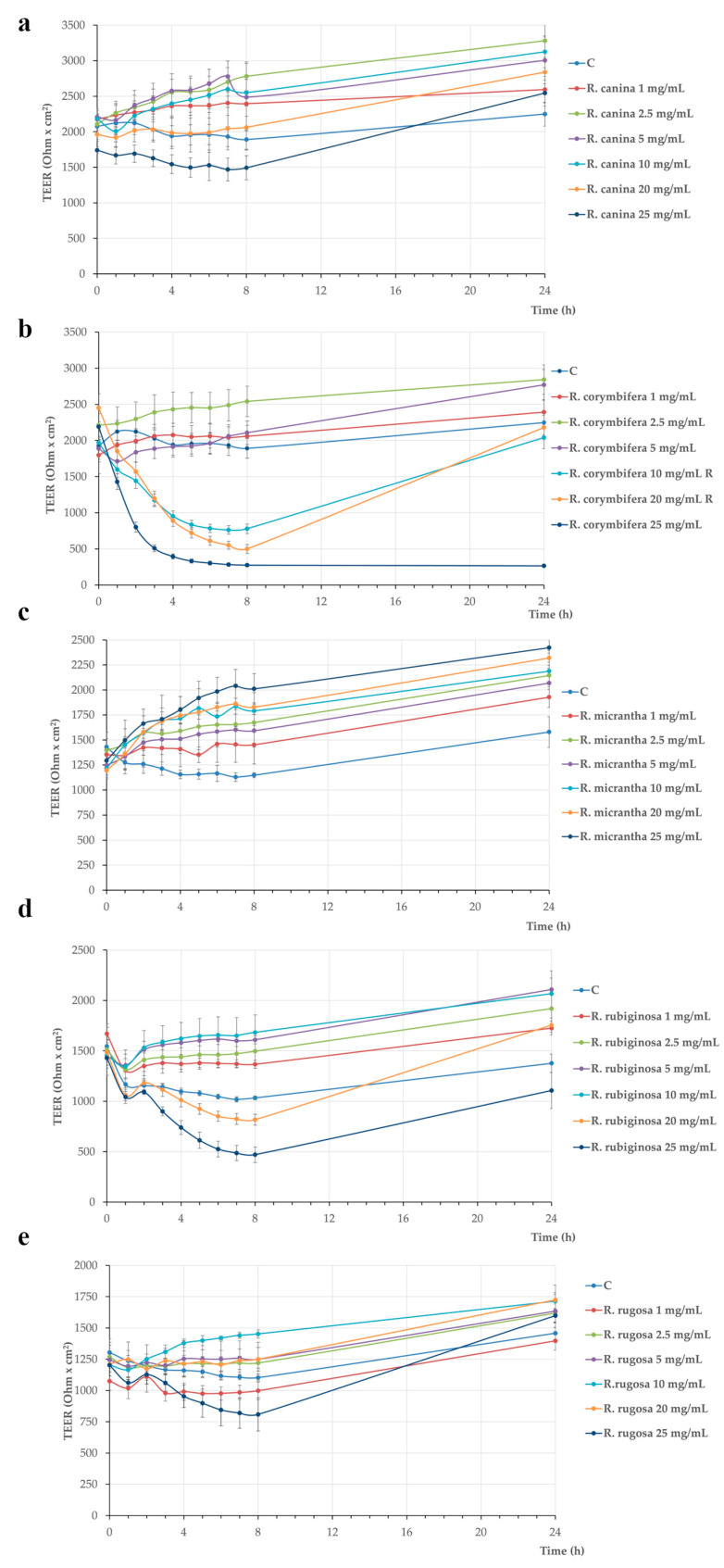
Rose hip extracts containing phenolic compounds effects on Caco-2 cells monolayer integrity, assayed by Transepithelial electrical resistance (TEER): *R. canina* (**a**); *R. corymbifera* (**b**); *R. micrantha* (**c**); *R. rubiginosa* (**d**); *R. rugosa* (**e**). Cells were untreated (Control, C) or treated with different extract concentrations (1–25 mg/mL). TEER values were recorded for up to 24 h and reported as Ohm × cm^2^. Values represent mean ± standard deviation, carried out in triplicate. R: recovery.

The results show that TEER values and corresponding Papp values of *R. canina*, *R. micrantha,* and *R. rugosa* were not affected by the different treatments for up to 24 h (Figure 3a,c,e and Table 4). Conversely, *R. rubiginosa* treatment induced a TEER decrease after 8 h, but the values were restored to control levels after 24 h (Figure 3d). Concerning *R. corymbifera*, the 25 mg/mL concentration induced a TEER drop already after 2 h, further decreasing between 3 and 5 h, up to the end of the experiment (Figure 3b). This TEER decrease was associated with a biologically relevant phenol red Papp increase, as the corresponding values were about 1.6 × 10^−6^ cm s^−1^, indicating that the tight junctions were open (Table 4, value in bold), as already described [33]. Finally, concerning 10 and 20 mg/mL *R. corymbifera*, the TEER drop was delayed at 5 h treatment compared to 25 mg/mL but did not reach the 25 mg/mL values (Figure 3b). To verify if such membrane damage could be reversible, the 10 and 20 mg/mL *R. corymbifera* samples underwent recovery; thus, the treatment medium was replaced by a fresh, complete medium from 8 to 24 h. TEER values at 24 h were similar to control, indicating that the cells were able to recover. These TEER results were confirmed by the data of phenol red paracellular passage, shown in Table 4. A large number of in vitro studies has shown that several polyphenols from different plant species are able to protect intestinal cells considering their numerous biological activities, including antimicrobial, antiproliferative, antioxidant and anti-inflammatory functions [34,35]. Several pieces of evidence indicate that quercitrin and catechin play a key role in membrane barrier protection and exert anti-inflammatory activity [26]. Although the extracts containing phenolic compounds from rose hips of *R. corymbifera* were the most abundant in catechin content, a TEER drop after treatment with the highest dose was observed in our study. Moreover, a reversible TEER drop after treatment of Caco-2 cells with high doses of *R. rubiginosa* extract could be detected. Even if in this extract catechin could not be detected, a common component in both species extract was quercitrin, that is, the 3-O-rhamnoside of the flavonol quercetin. Despite not sufficient data being available in the literature relating to the toxic effect of quercitrin and the related mechanisms still need to be elucidated [36], in the present study, both extracts presenting high levels of quercitrin induced a TEER drop after 8 h treatment; thus, a direct or indirect (due to other not detected molecules) role of quercitrin in membrane barrier damage can be speculated.

In order to determine how the intestinal tight junction status and the Caco-2 monolayer cells integrity (expressed as maintenance of high TEER) could be related to the levels of phenolic compounds in the rose hips, a partial least squares regression (PLSR) analysis was applied. Data collected on phenolic compounds constituted the independent X-block of variables, whereas the cell integrity attributes as measured through ∆TEER (i.e., the difference between the TEER measured at 8 h (t8) and the initial TEER (t0), for 25 mg/mL concentration of each of the five extracts containing phenolic compounds), represented the Y-variable.

**Figure 4:**
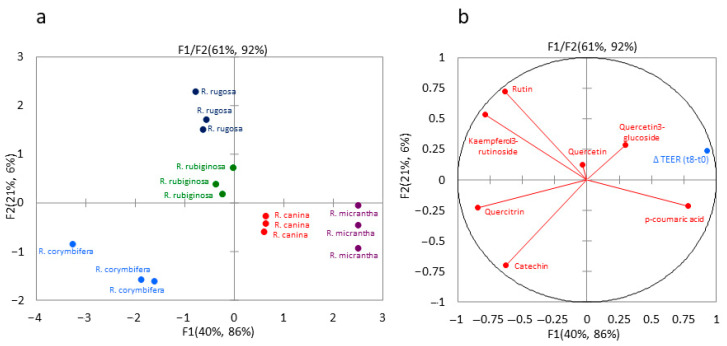
Weighed score (**a**) and loading (**b**) plots for group of phenolic compounds and ∆TEER values in rose hips of five different species. F: factor.

Although the analysis has been performed on a small amount of data, the PLSR model resulted in an R^2^ of 0.9899 and 0.9610 and a root mean square error (RMSE) of 88.0 and 185.0 in the predicted versus reference values in calibration and validation sets, respectively. The first two components of the PLSR predictive model explained 92% of the variance of the cell integrity as described by ∆TEER using 61% of phenolic compounds information provided by the used data set (Figure 4).

In the score plot, all five different rose species were clearly discriminated (Figure 4a), with *R. corymbifera* in the lower left quadrant (mainly associated with catechin and quercitrin, Figure 4b), *R. micrantha* and *R. canina* in the lower right quadrant (associated with p-coumaric acid), and *R. rugosa* and *R. rubiginosa* in the upper left quadrant (mainly driven by rutin and kaempferol 3-rutinoside). Moreover, the plot clearly shows that the ∆TEER was mainly predicted by the compounds that participate in forming the first component (F1 factor). In conclusion, this predictive model suggests a role of p-coumaric acid and quercetin 3-glucoside (directly or as being related to other non-analysed molecules or their secondary metabolites) in the integrity maintenance of the membrane barrier.

**Figure 5:**
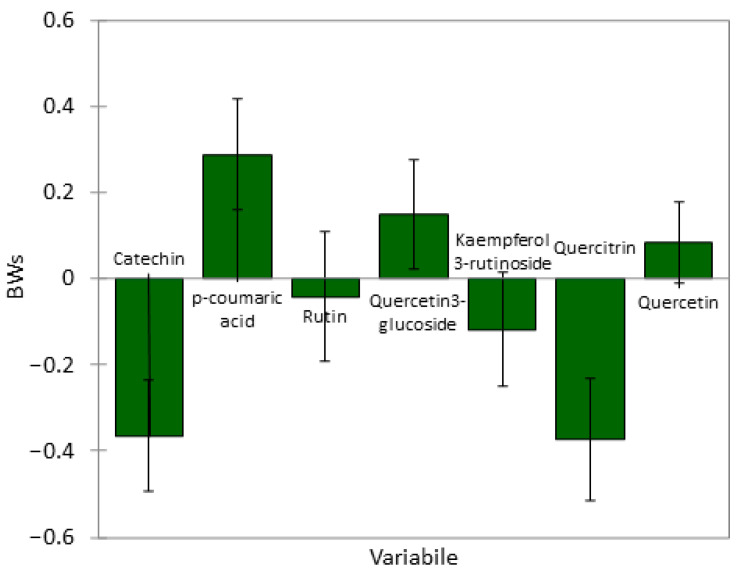
Weighed Regression Coefficients (BW) obtained from PLSR.

Figure 5 shows the Weighed Regression Coefficients (BW) obtained from the PLSR model, giving an indication of each phenolic compound’s contribution to the differences found in the observed membrane integrity maintenance effect. The observed BW clearly indicated that catechin and quercitrin, located in the lower-left quadrant (where *R. corymbifera* fell), were inversely correlated with intestinal cell integrity. On the other hand, quercetin 3-glucoside and p-coumaric acid positively participated in the definition of the predictive model. Noteworthy is the fact that the p-coumaric acid, although found in low concentrations (when present), showed high BW, implying its importance in the predictive power of this model regarding intestinal tight junction status and Caco-2 cell monolayer integrity.

## 3. Materials and Methods

### 3.1. Plant Material and Sampling

Cuttings propagated plants of *Rosa canina* L., *R. corymbifera* Borkh., *R. micrantha* Borrer ex Sm, *R. rubiginosa* L. and *R. rugosa* Thunb. were cultivated at the experimental farm of CREA-Research Centre for Plant Protection and Certification of Palermo (38°5′ N, 13°30′ E, 23 m above sea level), North-West Sicily, South Italy. The local climate was characterised by mild, moderately rainy winters and hot, dry summers. Rose plants were grown under outdoor conditions in single rows with a plant density of 10 plants m^−2^ and were irrigated only during summer through a drip irrigation system. Seventy mature rose hips were randomly (at different canopy heights) harvested at the same ripening stage (when hip colour was red) from ten-year-old plants (10 rose hips per plant) of the five selected species. A pool was then created for the hips of each rose species to study the biochemical differences among the species. A similar approach has been also used by Liaudanskas et al., 2021 [37]. After removing the seeds, the fresh rose hips were freeze-dried and ground into a very fine powder for biochemical analyses (phenolic compounds and carotenoids) by an analytical grinder (Janke & Kunkel, model A 10). Concerning the determination of vitamin C, it was evaluated on fresh rose hips, always after seed removal. All evaluations of biological activity were carried out at the CREA-Research Center for Food and Nutrition in Rome.

### 3.2. Morphological Characteristics

For each of the five considered species, the principal morphological characteristics of a rose leaf (number of leaflets/leaves, leaflet length, leaflet width, internode length), flower (petal length and width, hypanthium width), and hip (length, width, weight, and number of seeds/hips) were described. Fifty rose leaves, flowers, and hips per species were randomly selected from different levels of the canopy and measured. Leaflet length was measured with a tape ruler from the lamina tip to the point of intersection of the lamina and the rachis along the midrib of the lamina. Leaflet width was determined from end-to-end between the widest lobes of the lamina perpendicular to the lamina mid-rib. Internode length was determined as the distance between the intersection of two adjacent leaves and the stem. The flower petal length was measured with a tape ruler from the petal tip to its intersection with the hypanthium. Petal width was measured at its widest part, perpendicular to its length. The hip length was measured with a digital calliper from the pedicel to the calix; hip width was measured at the widest part of the rose hip, perpendicular to its length. Hip weight was determined with a technical balance (Gibertini Elettronica srl, Milan, Italy).

### 3.3. Determination of Vitamin C and Ascorbic Acid Content and Moisture Content

The moisture content was determined according to the AOAC official method. Vitamin C content, as the sum of ascorbic acid and dehydroascorbic acid, was determined by HPLC according to the method proposed by Tarrago-Trani et al. [38] with some modifications. To determine the dehydroascorbic acid and ascorbic acid becomes a double extraction of the sample, one using the extracting solution with the addition of the reducing agent, tris(2-carboxyethyl) phosphine TCEP 5 mM), and the other using only the extraction solution (metaphosphoric acid (MPA) 1.5%, EDTA 0.1 mg/mL). ACS grade ascorbic acid (AA) (>99% pure), metaphosphoric acid (MPA), and ethylenediaminetetraacetate disodium salt (EDTA) were purchased from Sigma-Aldrich (Saint Louis, MO, USA). TCEP, obtained from Thermo Scientific (Rockford, IL, USA), was used to reduce dehydroascorbic acid to ascorbic acid. About 2 g of fresh samples of rose hips were added, with 8 mL of the aqueous extraction solution and homogenised with an Ultra-Turrax at 8000 rpm for 4 min at 0 °C. The mixture was then centrifuged, and the supernatant was collected and cold-stored. The extraction was performed twice. The combined liquid extracts were filtered through a 0.45 µm syringe filter (RC membrane, Goettingen, Germany). The extraction was performed in duplicate. The determinations were carried out using an HPLC system (Agilent Technologies, Santa Clara, CA, USA) with an 1100 Series quaternary pump and diode array detector. The column used was a Synergi 4u Fusion-RP60A 150 × 4.6 mm (Phenomenex, Bologna, Italy), with Security Guard Cartridges AQ C18 4 × 30 mm. The column was thermostated to 30 °C; flow 0.8 mL/min for a total run time of 13 min; the injected volume was 10 µL. The mobile phase consisted of 0.02% (*w*/*v*) MPA pH 3.5 filtered with a Millipore apparatus. The spectrophotometric reading was at 240 nm, and the quantification was carried out using a calibration curve obtained by serial dilutions of pure ascorbic acid.

### 3.4. Determination of Phenolic Compounds Content

Approximately 2.5 g of lyophilised samples were extracted twice with 5 mL methanol acidified with 0.1% hydrochloric acid (according to the method of Medveckiene et al. [29]). The collected supernatants were increased to a final volume of 10 mL using methanol and then filtered with 0.45 µm syringe filters. To concentrate the sample, two 5 mL aliquots of the extracts were dried with nitrogen; one was resuspended in 1 mL methanol for HPLC and injected, while the other was used for biological activity assays (see paragraphs 3.6 and 3.8). The biochemical determinations were carried out using the HPLC system (Agilent Technologies, Santa Clara, CA, USA), with an 1100 Series quaternary pump and diode array detector. The column used was a Synergi 4 µm Polar-RP80 A, 150 × 4.6 mm with Security Guard Polar RP 4 × 3 m. The column was thermostated at 30 °C; the flow was set at 0.75 mL/min for a total run time of 60 min; the injected volume was 10 µL. The mobile phase used consisted of solution A (0.01 M H_3_PO_4_) and solution B (Methanol with 0.01 M H_3_PO_4_) with a binary gradient (for chromatographic conditions, see Baiamonte et al. [39]). The spectrophotometric reading was at 280-320-360 nm, and the quantification was carried out using a calibration curve obtained by serial dilutions of pure standards. Standards of quercetin 3-glucoside, quercetin, quercitrin, catechin, epicatechin, rutin, and kampferol 3-rutinoside were purchased from Merck (Darmstadt, Germany), whereas p-coumaric acid was purchased from Sigma-Aldrich (Saint Louis, MO, USA). HPLC-grade solvents were supplied by Carlo Erba Reagents (Milan, Italy). The extraction was performed in triplicate.

### 3.5. Determination of Carotenoid Content

Approximately 1 g of lyophilised sample was added to 500 mg CaCO_3_ and extracted with 20 mL tetrahydrofuran and methanol (THF/MetOH 1:1), added with 0.1% BHT (used to stabilise the solution). A 2,6-Di-tert-butyl-4-methylphenol (BHT) was purchased from Sigma-Aldrich (Saint Louis, MO, USA). Samples were stirred with a magnet for 30 min; the supernatant was collected in dark bowls and cold stored (according to the method of Al-Yafeai et al. [25], with small modifications). The extraction was repeated three times. Subsequently, the supernatants were filtered and evaporated with a rotary evaporator at a temperature of 30/35 °C to a volume of about 1 mL. The extracts were collected in 5 mL of the extraction solution THF/MetOH (1:1) and added to 0.1% BHT. Samples were filtered with 0.45 µm syringe filters before being injected. The extraction was performed in duplicate. The determinations were carried out using the HPLC system (Agilent Technologies, Santa Clara, CA, USA), with an 1100 Series quaternary pump and diode array detector; the column used was a YMC C 30 (5 µm, 4.6 × 250 mm), and a YMC basic VS precolumn (3 µm, 4.0 × 10 mm) was thermostated at 30°C; the flow rate was set at 1 mL/min for a total run time of 75 min; the injected volume was 10 µL. The mobile phase used consisted of solution A (methanol) and solution B (tert-butyl-methyl-ether, TBME) with a binary gradient. The spectrophotometric reading was at 450 nm for lutein and beta-carotene and at 470 nm for lycopene. The quantification of the compounds was carried out using a calibration curve obtained by serial dilutions of pure standards. Standards of lycopene, zeaxanthin, and lutein were obtained from Extrasynthèse (Genay, France), whereas β-carotene was purchased from Sigma-Aldrich (Saint Louis, MO, USA). HPLC-grade solvents were supplied by Carlo Erba Reagents (Milan, Italy).

### 3.6. Indicator Microorganism Strains and Agar Spot Test

To assess the potential antimicrobial of the phenolic compounds, the following microorganisms were used as indicator strains: *Salmonella enterica* serovar Typhimurium LT2 (DSMZ 18522 Braunschweig, Germany) and two *S. enterica* isolates from chicken belonging to two different serovars (Derby and Give), obtained by Istituto Zooprofilattico Sperimentale del Mezzogiorno (Portici, Naples, Italy); enterotoxigenic *Escherichia coli* K88 (ETEC, O149:K88ac), obtained by Istituto Zooprofilattico Sperimentale della Lombardia e dell’Emilia Romagna (Reggio Emilia, Italy); *Listeria monocytogenes* OH, *L. monocytogenes* CAL, *L. monocytogenes* SA and *L. innocua* 1770, provided by CREA-Research Centre for Animal Production and Aquaculture (Lodi, Italy), *Pseudomonas putida* WSC358, *P. putida* KT2240 and *P. fluorescens* BF13, kindly provided by Prof. Livia Leoni, Roma Tre University (Rome, Italy). Except for ETEC growing in Luria–Bertani Broth, Miller (DIFCO, Rodano (MI), Italy), all bacteria were routinely grown in Tryptone Soya Broth (Oxoid, Basingstoke, UK) at their optimal growth temperature, i.e., 30 °C for *Listeria* and *Pseudomonas* strains, and 37 °C for *Salmonella* and *Escherichia* strains. The five extracts containing phenolic compounds, dried under a steady stream of nitrogen, were resuspended in ethanol:water 80/20 *v*/*v*, as well as in PBS, and tested at two different concentrations (1 g/mL and 0.5 g/mL of the starting lyophilised material, for both alcoholic and aqueous extracts) for agar spot test, which was performed according to Zinno et al. [33]. Briefly, 2 μL of each extract were spotted onto 1.2% Tryptone Soya Agar (Oxoid) plates, previously seeded with each indicator strain (1 × 10^6^ CFU/mL), grown at log phase. Spotted plates were then incubated at 30 or 37 °C for 18–24 h, and inhibition halo (radius of the microbial growth inhibition zone) around the wells, when present, was measured in millimetres. Test efficacy was confirmed by adding 2 μL 50 μg/mL Ampicillin (Sigma-Aldrich, St Louis, MO, USA).

### 3.7. Intestinal Caco-2 Cell Culture

Caco-2 cells, obtained from INSERM (Paris, France), were used in passages 95 to 105. Caco-2 cells were routinely sub-cultured at low density (50% confluency, according to Natoli et al. [40] and maintained at 37 °C in a 5% CO_2_/95% air atmosphere at 90% relative humidity in DMEM containing 25 mM glucose, 3.7 g/L NaHCO_3_, and supplemented with 4 mM L-glutamine, 1% non-essential amino acids, 100 U/mL penicillin, 100 µg/mL streptomycin, and 10% heat-inactivated foetal calf serum (Euroclone, Milan, Italy). Cell culture media and reagents were purchased from Corning (Milan, Italy) unless otherwise stated. Cells were seeded at a density of 3.3 × 10^5^ cells/filter on polyethylene terephthalate permeable Transwell filters (Falcon^R^ 0.3 cm^2^ effective growth area, 0.4 μm pore size, Corning) and allowed to differentiate for 17–21 days.

### 3.8. Cell Monolayer Permeability Assessments

The *R. canina*, *R. corymbifera*, *R. micrantha*, *R. rubiginosa,* and *R. rugosa* hip extracts containing phenolic compounds resuspended in ethanol:water 80/20 *v*/*v* (1 g/mL) were diluted in serum-free cell culture medium and apically added at different concentrations (1-2.5-5-10-20-25 mg/mL) on differentiated Caco-2 cells, to evaluate their impact on membrane integrity. As a control, cell monolayers were also treated with 2% ethanol, corresponding to the amount contained in the higher concentration tested. To avoid possible interferences with foetal calf serum proteins, the complete cell culture medium was replaced by a serum-free medium 16 h before the assays.

The analysis of intestinal barrier integrity was performed according to Zinno et al. [33]. Transepithelial Electrical Resistance (TEER) values were expressed as Ohm (resistance) × cm^2^ (surface area of the filter) after subtracting the resistance value of the filter without cell monolayer. The TEER was checked the day before each experiment, and only cell monolayers with TEER values higher than 1300 Ohm × cm^2^ were used, as this value was identified in preliminary experiments as indicative of correct differentiation of Caco-2 cells. During the experiments, TEER was recorded every h for up to 8 h and then at 24 h. After 8 h treatment, some samples underwent recovery, obtained by replacing the treatment medium with a complete cell culture medium for up to 24 h. Cell permeability was also assessed at the end of treatments (24 h) by measuring the paracellular passage of the phenol red marker, defined as apparent permeability (Papp), expressed as cm s^−1^. Cell permeability was also assessed at the end of treatments (24 h) by measuring trans-epithelial passage of the paracellular marker phenol red [41]. Phenol red Papp values below 1 × 10^−6^ cm s^−1^ were considered indicative of intact monolayers [42]. Thus, this value was set up as a threshold, irrespectively of statistical significance among samples. Experiments were performed in triplicate.

### 3.9. Statistical Analysis

The statistical significance of the differences was evaluated by one-way ANOVA, followed by post hoc Tukey’s Multiple Comparisons Test (HSD) test, after verifying normality and homoscedasticity by Shapiro–Wilk’s and Levene’s tests, respectively. For morphological data, Duncan’s Multiple Range test was performed. All statistical tests were run with Microsoft Office Excel 2011 upgraded with XLSTAT (ver. 4 March 2014). Mean values with different superscript letters significantly differ, and statistical significance was set at *p* < 0.05.

A Multivariate partial least squares regression (PLSR) with a group of phenolic compounds analysed in the rose hips as predictive variables (X) and their effects on Caco-2 cell monolayer integrity (reported as ΔTEER = TEER t8h–TEER t0 for 25 mg/mL concentration of each of the five extracts containing phenolic compounds) as dependent variables (Y) was used to unravel possible relationships between the two blocks. Data were processed using the NIPALS algorithm and normalised to equalise their potential influence in the model (Unscrambler, v. 10.2, CAMO Software AS, Norway). No rotation method was implemented, while a cross-validation procedure to determine the maximum number of significant dimensions was applied.

## 4. Conclusions

In the present study, a comparative analysis of five rose hip species (*R. canina*, *R. corymbifera*, *R. micrantha*, *R. rubiginosa* and *R. rugosa*) cultivated in Sicily under the same environmental conditions was carried out. Albeit these data deserve further investigations, to the best of our knowledge, this is the first study comparing five different rose species through the analysis of morphological aspects of leaves, flowers, hips, biochemical profile (content of vitamin C, carotenoids, and phenolic compounds), and biological activities (the antimicrobial activity and the effect on epithelial integrity in an in vitro model of intestinal cells).

The multivariate analysis (Partial Least Squares Regression) allowed us to successfully discriminate the five *Rosa* species and to apply a predictive model to correlate epithelial integrity and bioactive phenolic content in an intestinal in vitro cell model, indicating a possible direct and/or indirect role of catechin, quercitrin, and p-coumaric acid. Although few data are available in the literature on the toxicity of high concentrations of phenolic compounds on the intestinal monolayer, this predictive model suggests further insights into the molecular mechanisms underlying such effects.

Although this work has some limitations due to the small number of analysed parameters and the type of sampling that reduces intra-species variability information, these findings can play an important role in the future exploitation of rose hips grown in Sicily as new functional foods and as new sources of bioactive compounds and natural antioxidants of plant origin with beneficial health effects to be used as supplements in food and nutraceutical fields.

## Figures and Tables

**Table 1:** Morphological characteristics of leaf, flower, and hip from the five studied rose species.

	*R. canina*	*R. corymbifera*	*R. micrantha*	*R. rubiginosa*	*R. rugosa*
**Leaflets/leaf (n.)**	6.6 ± 0.16 ^ab^	6.0 ± 0.23 ^b^	5.8 ± 0.17 ^b^	5.9 ± 0.09 ^b^	8.2 ± 0.18 ^a^
**Leaflet length (cm)**	5.1 ± 0.16 ^a^	3.9 ± 0.12 ^ab^	4.4 ± 0.13 ^ab^	2.3 ± 0.10 ^b^	3.8 ± 0.12 ^ab^
**Leaflet width (cm)**	3.0 ± 0.06 ^a^	2.2 ± 0.06 ^b^	2.7 ± 0.07 ^a^	1.7 ± 0.07 ^b^	2.7 ± 0.07 ^a^
**Internode length (cm)**	2.6 ± 0.11 ^ab^	3.7 ± 0.38 ^a^	2.6 ± 0.12 ^ab^	3.0 ± 0.12 ^a^	1.6 ± 0.09 ^b^
**Petal length (cm)**	2.8 ± 0.05 ^b^	2.9 ± 0.03 ^b^	2.6 ± 0.07 ^b^	2.9 ± 0.04 ^b^	4.5 ± 0.06 ^a^
**Petal width (cm)**	2.6 ± 0.01 ^b^	2.3 ± 0.04 ^b^	2.4 ± 0.02 ^b^	2.6 ± 0.08 ^b^	4.7 ± 0.07 ^a^
**Hypanthium width (cm)**	0.5 ± 0.04 ^b^	0.5 ± 0.02 ^b^	0.6 ± 0.03 ^b^	0.6 ± 0.05 ^b^	1.0 ± 0.09 ^a^
**Hip length (cm)**	2.0 ± 0.11 ^a^	1.9 ± 0.15 ^a^	1.8 ± 0.17 ^a^	1.9 ± 0.13 ^a^	2.1 ± 0.62 ^a^
**Hip width (cm)**	1.2 ± 0.33 ^b^	1.5 ± 0.26 ^a^	1.4 ± 0.22 ^ab^	1.6 ± 0.25 ^a^	1.8 ± 0.43 ^a^
**Hip weight (g)**	1.5 ± 0.03 ^b^	2.1 ± 0.07 ^b^	1.8 ± 0.05 ^b^	1.7 ± 0.08 ^b^	3.0 ± 0.33 ^a^
**Seeds/hip (n.)**	21.6 ± 0.90 ^b^	20.4 ± 0.66 ^b^	13.2 ± 0.83 ^c^	18.4 ± 0.78 ^b^	42.5 ± 0.19 ^a^

In each row, values are means ± standard error. Means were calculated on fifty leaves, flowers, and hips randomly collected from each species. Means with different letters significantly differ (*p* < 0.05, Duncan’s Multiple Range test).

**Table 2:** Phenolic compound concentrations (mg/g of dry matter) in the five rose hip species grown in Sicily.

Rose Species	Catechin mg/g	p-Coumaric Acid mg/g	Rutin mg/g	Quercetin 3-Glucoside mg/g	Kaempferol 3-Rutinoside mg/g	Quercitrin mg/g	Quercetin mg/g
***R. canina***	0.181 ± 0.008 ^b^	_ ^b^	0.067 ± 0.004 ^b^	0.044 ±0.003 ^a^	0.019 ±0.001 ^bc^	0.003 ±0.000 ^b^	tr ^a^
***R. corymbifera***	0.419 ± 0.016 ^a^	tr^b^	0.074 ±0.017 ^b^	0.010 ± 0.007 ^c^	0.033 ± 0.009 ^ab^	0.040 ±0.012 ^a^	0.002 ± 0.001 ^a^
***R. micrantha***	0.006 ± 0.000 ^c^	0.002 ±0.000 ^a^	0.025 ± 0.001 ^c^	0.017 ± 0.001 ^bc^	0.008 ± 0.001 ^c^	0.005 ± 0.000 ^b^	0.002 ± 0.000 ^a^
***R. rubiginosa***	_ ^c^	_ ^b^	0.093 ± 0.007 ^ab^	0.027 ± 0.004 ^b^	0.027 ± 0.010 ^b^	0.021 ± 0.013 ^ab^	0.001 ± 0.000 ^a^
***R. rugosa***	tr ^c^	_ ^b^	0.125 ± 0.024 ^a^	0.027 ± 0.008 ^b^	0.044 ± 0.001 ^a^	0.015 ± 0.001 ^b^	0.001 ± 0.000 ^a^
***Pr > F***	**<0.0001**	**<0.0001**	**<0.0001**	**<0.0001**	**<0.0001**	**0.001**	**0.092**
**Significant**	**Yes**	**Yes**	**Yes**	**Yes**	**Yes**	**Yes**	**No**

Results are expressed as mean ± standard deviation of three independent extractions. Within each column, differences were evaluated by one-way ANOVA and Tukey’s Multiple Comparisons Test (HSD) 95%. Values with different letters significantly differ (*p* < 0.05). tr: trace. -: not detected.

**Table 3:** Carotenoid content (µg/g dry matter) in the five rose hip species grown in Sicily.

Rose Species	Lutein µg/g	Zeaxanthin µg/g	β-Carotene µg/g	Lycopene µg/g
***R. canina***	5.32 ± 0.33 ^b^	1.42 ± 0.05 ^d^	15.45 ± 0.73 ^b^	19.44 ± 0.17 ^b^
***R. corymbifera***	5.63 ± 0.83 ^b^	3.16 ± 0.77 ^bc^	68.29 ± 10.28 ^a^	32.88 ± 9.33 ^ab^
***R. micrantha***	5.94 ± 0.08 ^b^	4.35 ± 0.18 ^b^	51.99 ± 2.34 ^a^	43.96 ± 4.58 ^ab^
***R. rubiginosa***	4.71 ± 0.01 ^b^	2.47 ± 0.01 ^cd^	26.99 ± 0.61 ^b^	80.96 ± 28.25 ^a^
***R. rugosa***	10.79 ± 0.28 ^a^	11.62 ± 0.28 ^a^	59.09 ± 1.13 ^a^	53.63 ± 4.67 ^ab^
***Pr > F***	**<0.0001**	**<0.0001**	**<0.0001**	**0.04**
**Significant**	**Yes**	**Yes**	**Yes**	**Yes**

Results are expressed as mean ± standard deviation of two independent extractions. Within each column, differences were evaluated by one-way ANOVA followed by Tukey’s Multiple Comparisons Test (HSD) 95%. Values with different letters significantly differ (*p* < 0.05).

**Table 4:** Phenol red apparent permeability (Papp) measured in differentiated Caco-2 cells after 24 h treatment with the five rose hip extracts containing phenolic compounds at 10, 20, and 25 mg/mL.

Sample	Concentration (mg/mL)	Papp × 10^−6^ (cm s^−1^)
**C**		0.21 ± 0.12
***R. canina***	10	0.24 ± 0.03
	20	0.16 ± 0.01
	25	0.17 ± 0.01
***R. corymbifera***	10	0.13 ± 0.01
	20	0.12 ± 0.04
	**25**	**1.61** **±** **0.03**
***R. micrantha***	10	*nd*
	20	*nd*
	25	*nd*
***R. rubiginosa***	10	0.18 ± 0.03
	20	0.19 ± 0.03
	25	0.25 ± 0.02
***R. rugosa***	10	*nd*
	20	*nd*
	25	*nd*

Phenol red Papp was measured at 24 h, and values are means ± standard deviation and reported as cm s^−1^. The experiments were carried out in triplicate. C: Control (untreated cells); *nd*: not determined.

## Data Availability

The data presented in this study are available upon request from the corresponding authors.

## Supplementary Materials

The following supporting information can be downloaded at: https://www.mdpi.com/article/10.3390/plants13010053/s1, Table S1: % Moisture content in the five rose hip species grown in Sicily; Table S2: Antimicrobial activity of extracts containing phenolic compounds against various pathogen indicator strains (spot test).
