# Supplementary material for: Comparative Study of Bioactive Compounds and Biological Activities of Five Rose Hip Species Grown in Sicily
Source: Plants (Basel). 2023 Dec 23;13(1):53. doi: 10.3390/plants13010053 (PMC10780848; doi:10.3390/plants13010053)
Supplement: Supplementary file 1 [file plants-13-00053-s001.zip › Table S1.pdf]

Table S1. % Moisture content in the five rose hip species grown in Sicily.

Rose species	*% Moisture*
R. canina	59.30 ± 1.04[c]
R. corymbifera	63.70 ± 1.27[bc]
R. micrantha	66.71 ± 0.75[b]
R. rubiginosa	64.20 ± 0.38[b]
R. rugosa	75.10 ± 1.70[a]
Pr > F	0.000
Significant	Yes

Results are expressed as mean ± standard deviation of two independent measurements. Values with different letters significantly differ ($p < 0.05$). One-Way ANOVA and Tukey's Multiple Comparisons Test (HSD) 95%.
