# Supplementary material for: Comparative Study of Bioactive Compounds and Biological Activities of Five Rose Hip Species Grown in Sicily
Source: Plants (Basel). 2023 Dec 23;13(1):53. doi: 10.3390/plants13010053 (PMC10780848; doi:10.3390/plants13010053)
Supplement: Supplementary file 1 [file plants-13-00053-s001.zip › Table S2.pdf]

Table S2. Antimicrobial activity of extracts containing phenolic compounds against various pathogen indicator strains (spot test).

**Strain**	***Inhibition Halo Radius***															
	R. canina			***R. corymbifera***			***R. micrantha***			***R. rubiginosa***			***R. rugosa***			**Ampicillin**
	Ethanol:water		***PBS***	***Ethanol:water***		***PBS***	***Ethanol:water***		***PBS***	***Ethanol:water***		***PBS***	***Ethanol:water***		***PBS***	
	0.5 g/mL	***1 g/mL***	***1 g/mL***	***0.5 g/mL***	***1 g/mL***	***1 g/mL***	***0.5 g/mL***	***1 g/mL***	***1 g/mL***	***0.5 g/mL***	***1 g/mL***	***1 g/mL***	***0.5 g/mL***	***1 g/mL***	***1 g/mL***	***50 µg/mL***
L. monocytogenes OH	-	-	-	-	-	-	-	-	-	-	-	-	-	-	-	+
L. monocytogenes SA	-	-	-	-	-	-	-	-	-	-	-	-	-	-	-	+
L. monocytogenes CAL	-	-	-	-	-	-	-	-	-	-	-	-	-	-	-	+
L. innocua 1770	-	-	-	-	-	-	-	-	-	-	-	-	-	-	-	+
S. enterica Typhimurium LT2	-	-	-	-	-	-	-	-	-	-	-	-	-	-	-	+
S. enterica Give	-	-	-	-	-	-	-	-	-	-	-	-	-	-	-	+
S. enterica Derby	-	-	-	-	-	-	-	-	-	-	-	-	-	-	-	+
E. coli (ETEC) K88	-	-	-	-	-	-	-	-	-	-	-	-	-	-	-	+
P. putida WSC358	-	-	-	-	-	-	-	-	-	-	-	-	-	-	-	+
P. putida KT2240	-	-	-	-	-	-	-	-	-	-	-	-	-	-	-	+
P. fluorescens BF13	-	-	-	-	-	-	-	-	-	-	-	-	-	-	-	+

The extracts containing phenolic compounds were resuspended in both ethanol:water 80:20 (0.5 and 1 g/mL) and in aqueous solution (PBS, 1 g/mL).
Antimicrobial activity was considered high (+) when the value of the inhibition halo radius was greater than 4.3 mm and absent (-) when the halo was not detectable.
